# Reply to: False conflict and false confirmation errors are crucial components of AI accuracy in medical decision making

**DOI:** 10.1038/s41467-024-50954-1

**Published:** 2024-08-13

**Authors:** Christoph Wies, Katja Hauser, Titus J. Brinker

**Affiliations:** 1 Digital Biomarkers for Oncology Group, German Cancer Research Center (DKFZ), Heidelberg, Germany; 2 Medical Faculty, University Heidelberg, Heidelberg, Germany

**Keywords:** Statistics, Cancer prevention

**replying to** R. Rosenbacke et al*. Nature Communications* 10.1038/s41467-024-50952-3 (2024)

The purpose of our publication “Dermatologist- explainable AI enhances trust and confidence in diagnosing melanoma” was to build and evaluate an explainable AI (XAI) model to distinguish between melanomas and nevi^1^. The model was intended as a prototype of an assistance system for clinicians and thus designed to be able to explain its decisions in a dermatologist-understandable way. In addition to the development of the XAI, we conducted a reader study with clinicians to quantify their interaction with our XAI with regard to the influence on the clinicians’ diagnostic accuracy, their confidence in their own diagnoses and their trust in the assistance systemlike. The reader study was conducted in different phases, so that the clinicians diagnosed the same lesions at different points in time with at least two weeks in between with different levels of AI support to increase comparability. Rosenbacke, Melhus and Stuckler focused in their Matters Arising on three error types that occur in the field of human-AI-interactions and on sub-group investigations based on physicians’ performance^2^; an important topic in human-AI interaction tasks overall^3,4^.

We fully agree with Rosenbacke and colleagues that the investigation of these three errors and the performance-based sub-group analysis are highly relevant points for the introduction of (X)AI into clinical practice. They mention three types of errors that can occur: (i) false confirmation error—when the physician and the AI agree but both are wrong; (ii) false conflict error—when the physician is correct, AI is incorrect, and the physician changes diagnosis (which is a particularly difficult case from an ethical perspective^5^); and (iii) true conflict error—when the physician is incorrect but AI is correct, and the physician overrides the correct AI diagnosis. From our point of view, those three errors are very important to investigate, but not able to provide a complete picture. Thus, we propose introducing one additional error type and four additional scenarios leading to correctly diagnosed cases. The errors Rosenbacke and colleagues did not mention are the (iv) true confirmation errors—when both the physician and the AI diagnosis are correct, but the physician subsequently switches to an incorrect diagnosis. This occurred in 3.9% of cases in our dataset and might be caused by unrealistic explanations. Furthermore, we argue that we must also consider the four correctly diagnosed cases to paint a complete picture. These are: (a) correct true confirmation cases—when both the AI and the physician are correct and the physician does not change their diagnosis; (b) correct true conflict cases—when the AI is correct and the physician is wrong, but the physician accepts the AI decision; (c) correct false confirmation cases—when both the AI and the physician’s initial diagnoses are wrong, but the physician changes their diagnosis when receiving an incorrect AI suggestion; and (d) correct false conflict cases—when the AI is wrong, the clinician is correct and the clinician overrides the incorrect AI decision. Especially when investigating the individual errors for different subgroups, it is necessary to take all eight cases into account. All the mentioned scenarios are summarized in Table 1, Subtable A) which also contains the scenario identifiers (i–iv and a–d). It should be noted that the AI’s and the clinicians’ correctness are independent, since the clinicians delivered their initial diagnosis without AI advice. To conduct the sub-group analysis, we defined the 25%-quantile in physicians’ accuracy as the threshold for the worst performers and the AI’s accuracy (80.4%) as the threshold for the best performers respectively, as suggested by Rosenbacke and colleagues^2^. We report the absolute numbers for all 8 cases in Table 1 for the whole available dataset (Subtable B), the best performers (Subtable C) and the worst performers (Subtable D). It should be noted that the relative performance of a clinician is not trivially discernible in a clinical setting. However, it might be correlated with years of experience or the weekly load of lesions seen by the clinician.

**Table 1:** Confusion matrix of correctness of human–XAI-collaboration with regard to the physician switching or keeping the diagnosis after getting XAI advice

	Physician correct		Physician wrong	
**A**	Theoretical concept			
XAI correct	True confirmation error **iv**	True confirmation (correct) **a**	True conflict (correct) **b**	True conflict error **iii**
XAI wrong	False conflict error **ii**	False conflict (correct) **d**	False confirmation (correct) **c**	False confirmation error **i**
	*Physician changes with AI/XAI advice*	*Physician stays with AI/XAI advice*	*Physician changes with AI/XAI advice*	*Physician stays with AI/XAI advice*
**B**	All physicians (*n* = 109)			
XAI correct	58 (3.8%)	**795** (52.7%)	**199** (13.2%)	160 (10.6%)
XAI wrong	64 (4.2%)	**82** (5.4%)	**19** (1.3%)	131 (8.7%)
	*Physician changes with XAI advice*	*Physician stays with XAI advice*	*Physician changes with XAI advice*	*Physician stays with XAI advice*
**C**	Best physicians (*n* = 15)			
XAI correct	8 (3.9%)	**145** (70.7%)	**7** (3.4%)	6 (2.9%)
XAI wrong	12 (5.9%)	**14** (6.8%)	**1** (0.5%)	12 (5.9%)
	*Physician changes with XAI advice*	*Physician stays with XAI advice*	*Physician changes with XAI advice*	*Physician stays with XAI advice*
**D**	worst physicians (*n* = 20)			
XAI correct	4 (1.5%)	**99** (36.4%)	**52** (19.1%)	52 (19.1%)
XAI wrong	6 (2.2%)	**18** (6.6%)	**4** (1.5%)	37 (13.6%)
	*Physician changes with XAI advice*	*Physician stays with XAI advice*	*Physician changes with XAI advice*	*Physician stays with XAI advice*

Subtable A describes the theoretical concept in which cases can fall into one of 8 scenarios: 4 errors (i–iv) and 4 correct predictions (a–d). Subtable B–D correspond to the publicly available data from Chanda and colleagues^1^. Here B refers to the whole set of participating clinicians, whereas C refers to the best performing clinicians and D to the worst performing clinicians. For Subtable B–D.

*In this table, bold numbers mark numbers with correct diagnoses after taking AI advice into account.

*In this table, underlined numbers denote numbers with incorrect diagnoses after taking AI advice into account.

We agree with most of the findings Rosenbacke and colleagues developed, but not with all of their points. We investigated the concordant as well as the discordant findings based on the numbers shown in Table 1, Subtable B–D. Based on these numbers, we investigated the findings of Rosenbacke and colleagues: “The best clinicians lose accuracy”—We verify this statement with Table 1, where we show that for the best performers overall 26 diagnoses were incorrect without AI support (this can be assessed by adding up all numbers in the “physician wrong”-column in Table 1C, but the number of incorrect diagnoses increased to 38 with AI support (this can be assessed by adding up all underlined numbers within Table 1C. “The most common and discussed error occurs when physicians tend to override a correct AI diagnosis in cases of true conflict error. Previous studies found that this arises from distrust in the AI’s ‘black box’ logic”—This statement can be verified by comparing Table 1B and Table S1. In Table 1B, we show that 160 out of 359 true conflict cases ended up in an error with XAI support. Table S1 shows that 170 out of those 359 end up in an error with only AI support. Thus, 10 true conflict errors could be prevented by adding explanations to the black box model. “(…) AI, for the lowest performing clinicians, helped stamp out true conflict errors”—This statement can be disproved and underlines, why the correct cases are important to take into account alongside the errors: if we investigate all 359 true conflict cases, they divide into 160 true conflict errors and 199 correct true conflict cases. Thus, we end up with an error rate of 44.6% investigating only the true conflict cases. For the worst performing clinicians, the 104 true conflict cases divide into 52 true conflict errors and 52 correct true conflict cases, which lead to an error rate of 50% for the true conflict cases.

In conclusion, it is of critical importance to investigate the different errors when physicians make decisions with AI support especially with respect to the human expertise level^4,6^. But besides the different error types, it is also important to take the correctly classified cases into account to get a complete picture of the situation. Further research is needed to investigate why those errors occur, whether certain subgroups of clinicians are at particular risk of committing certain error scenarios, and how this can be prevented. One potential approach is taking AI uncertainty into account by giving clinicians AI advice^7^. Of special interest are furthermore cases, where AI and clinician are initially correct, but the clinician overwrites both, his original diagnosis and the XAI’s advice (Table 1C; scenario iv), which need to be investigated carefully.

## Reporting Summary

Further information on research design is available in the Nature Portfolio Reporting Summary linked to this article.

### Supplementary Information

Table S1

Reporting Summary

## Data Availability

The code and data used generated in this study have been deposited in the codeocean-capsule under accession code https://codeocean.com/capsule/6210582/tree/v1.
