# Supplementary material for: Reply to: False conflict and false confirmation errors are crucial components of AI accuracy in medical decision making
Source: Nat Commun. 2024 Aug 13;15:6897. doi: 10.1038/s41467-024-50954-1 (PMC11322447; doi:10.1038/s41467-024-50954-1)
Supplement: Supplementary file 1 — Table S1 [file 41467_2024_50954_MOESM1_ESM.pdf]

Supplements

Table S1: *Confusion matrix of correctness of human-AI-collaboration with regard to the physician switching or keeping the diagnosis after getting AI advice without explanation. This table refers to the set of all physicians, bold numbers are correct after taking AI advice into account, underlined numbers are incorrect after taking AI advice into account.*

	physician correct		physician wrong	
	all physicians (n=109) but only with AI-advise, no XAI			
AI correct	65	**788**	**189**	170
AI wrong	50	**96**	**16**	134
	physician changes with AI advice	*physician stays with AI advice*	*physician changes with AI advice*	*physician stays with AI advice*
